# Re-polarizing Myeloid-derived Suppressor Cells (MDSCs) with Cationic Polymers for Cancer Immunotherapy

**DOI:** 10.1038/srep24506

**Published:** 2016-04-14

**Authors:** Wei He, Pei Liang, Guangxing Guo, Zhen Huang, Yiming Niu, Lei Dong, Chunming Wang, Junfeng Zhang

**Affiliations:** 1 State Key Laboratory of Pharmaceutical Biotechnology, NJU Advanced Institute for Life Sciences (NAILS), School of life sciences, Nanjing University, 163 Xianlin Avenue, Nanjing 210093, China; 2 State Key Laboratory of Quality Research in Chinese Medicine, Institute of Chinese Medical Sciences, University of Macau, Taipa, Macau SAR; 3 Jiangsu Provincial Laboratory for Nano-Technology, Nanjing University, Nanjing, China

## Abstract

Our evolving understandings of cell-material interactions provide insights for using polymers to modulate cell behaviour that may lead to therapeutic applications. It is known that in certain cancers, myeloid-derived suppressor cells (MDSCs) play vital roles in promoting tumour progression, chiefly because of their ‘alternatively activated’ (or M2) phenotype that orchestrates immunosuppression. In this study, we demonstrated that two cationic polymers – cationic dextran (C-dextran) and polyethyleneimine (PEI) – could directly remodel these cells into an anti-tumour, ‘classically activated’ (or M1) phenotype, thereby stimulating these cells to express tumouricidal cytokines, reactivating the T cell functions, and prolonging the lifespan of the mice model. Our investigations with knock-out mice further indicate that the functions of these cationic polymers require the involvement of toll-like receptor 4-mediated signalling. Taken together, our study suggests that these cationic polymers can effectively and directly re-polarize MDSCs from an immunosuppressive characteristic to an anti-tumour phenotype, leading to successful restoration of immune surveillance in the tumour microenvironment and elimination of tumour cells. Our findings may have immediate impact on further development of polymer-based therapeutics for cancer immunotherapy.

Cancer immunosuppression is the reduction of cellular and molecular activities of the immune system against tumour, leading to cancer progression and failure of immunotherapeutic attempts^1^,^2^. Various cell types play diverse roles in orchestrating immunosuppression in different cancers^3^. Emerging evidences suggest that myeloid-derived suppressor cells (MDSCs), a heterogeneous group of immature myeloid cells, play a central role in promoting immunosuppression in certain cancer types such as breast, pancreatic, colon and non-small cell lung cancers^4^,^5^,^6^,^7^,^8^,^9^. These cells, identified by the co-expression of Gr-1 and CD11b in mice^10^,^11^, lack the ability to differentiate into more specialized innate immune cells such as macrophages^12^,^13^. But like macrophages, MDSCs can also be classified into a classically (M1) or an alternatively (M2) activated phenotype^14^,^15^,^16^. M1-type MDSCs exhibit anti-tumour activities by secreting interleukin-12 (IL-12), tumour necrosis factor (TNF-α) and nitric oxide (NO); whereas M2-polarized MDSCs suppress T lymphocytes-mediated tumour-killing responses through the expression of interleukin-10 (IL-10), transforming growth factor (TGF-β) and arginase^17^,^18^,^19^. In the likes of breast cancer, MDSCs are predominantly polarized into M2 type. They spread across many tissues ranging from bone marrow to the spleen to the blood, and pose higher impact than TAMs on tumour progression^20^,^21^. Successful reversion of the MDSC’s polarization from M2- to M1-type may provide new insights for improving the efficacy of cancer immunotherapy.

Cationic polymers may possess the potential to re-polarize MDSCs for two reasons. Firstly, several studies have demonstrated that modulation of the toll-like receptors (TLRs) signalling in MDSCs could re-activate immunosurveillance against tumour and thereby enhance immunotherapy^22^,^23^,^24^,^25^. Secondly, cationic polymers, which have been used as gene-delivering carriers for decades^26^, could directly regulate the functions of immune cells – possibly through TLRs^27^,^28^. Our previous studies reported that two cationic polymers, namely cationic dextran (C-dextran) and polyethyleneimine (PEI), stimulated macrophages to produce immune-activating cytokine IL-12 via toll-like receptor 4 (TLR4) signalling^27^, and re-directed tumour-associated macrophages (TAM) from a immunosuppressive to tumour-killing phenotype^29^. Therefore, we hypothesized that cationic polymers could re-polarize MDSCs via TLR4 signalling, consequently triggering desired immune responses and restoring cancer immunosurveillance.

To validate this, in the present study, we investigated the influence of the two cationic polymers on the phenotypes and functions of MDSCs both *in vitro* and *in vivo*, with particular emphases on the role of TLR4 in such process and evaluation of these polymers as potential polymer therapeutics. We demonstrated for the first time that these cationic polymers could reverse the M2-like phenotype of MDSCs and reduce their immunosuppressive functions via TLR4 signalling.

## Results

### Cationic materials program the phenotypic polarization of MDSCs

**Figure 1:**
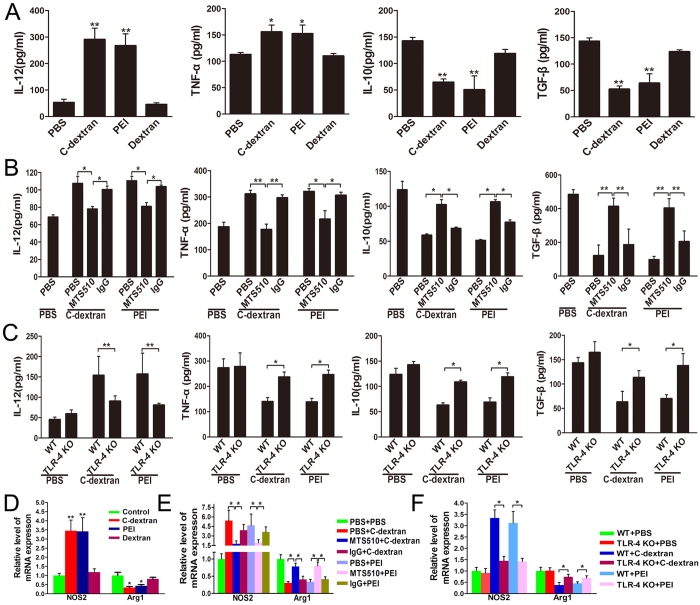
Effect of the cationic polymers on the phenotype of tumour-infiltrating MDSCs *in vitro*. (**A–C**) The concentrations of IL-12, TNF-α, IL-10 and TGF-β in the supernatant of sorted MDSCs which were (**A**) treated with C-dextran, PEI or dextran (25 μg mL^−1^) for 6 hours; (**B**) pre-treated with PBS, 20 μg mL^−1^ TLR-4-neutralising antibody (MTS510) or 20 μg mL^−1^ control IgG (Rat IgG2a, Kappa) for 1 hour, and then treated with C-dextran or PEI (25 μg mL^−1^) for 6 hours and (**C**) from WT or TLR-4^−/−^ tumour bearing-mice and treated with C-dextran or PEI (25 μg mL^−1^) for 6 hours were determined by ELISA; (**D–F**) Transcriptional levels of iNOS2 and Arg1 in MDSCs from each treatment cohort were analysed by qRT-PCR. The data represent 3–4 independent experiments (n* ***= **3–4, ^*^P ≤ 0.05 and ^**^P ≤ 0.01 versus saline).

We treated MDSCs with dextran, C-dextran or PEI, and then determined the concentrations of IL-12, TNF-α, IL-10 and TGF-β in the supernatant and the mRNA levels of iNOS and Arg I. We found that both cationic polymers, C-dextran and PEI, stimulated the cells to secrete IL-12 and TNF-α (Fig. 1A) and increased the transcriptional level of iNOS (Fig. 1D). In contrast, these polymers suppressed the expression of IL-10 and TGF-β (Fig. 1A) and decreased Arg I expression (Fig. 1D) at mRNA level. To determine the percentage of MDSCs re-polarized from M2- to M1-like phenotype by the cationic polymers, we analysed by flow cytometry the ability of these cells to produce IL-12 and TNF-α. We found that the cationic polymers could induce more MDSCs to produce IL-12 (Supplementary Fig. 1A,C) and TNF-α (Supplementary Fig. 1B,D), in comparison with those cells treated with Dextran or PBS. These data confirmed that MDSCs in the tumour tissue were predominantly (80–90%) M2-polarized, and demonstrated that the cationic polymers could effectively switch around 30% of total MDSCs from M2- to M1-like phenotype – which was 2–3 folds of the original percentage of M1 fraction.

Because we previously found that cationic polymers could induce IL-12 secretion in a TLR-4 dependent manner^27^, we examined whether cationic polymers would switch the phenotype of MDSCs via the same mechanism. We found that MTS510, a TLR-4-blocking antibody, completely abolished the effect of these cationic polymers on MDSCs re-polarization, as no difference was found between this group and untreated MDSCs in the levels of IL-12, TNF-α, IL-10, TGF-β, iNOS and Arg I (Fig. 1B,E). Similarly, compared with WT MDSCs, the MDSCs collected from TLR-4^−/−^ (KO) mice had no significant change in the levels of those specific markers after cationic polymers treatment (Fig. 1C,F).

### MDSCs lose their suppressive function after cationic polymer stimulation

**Figure 2:**
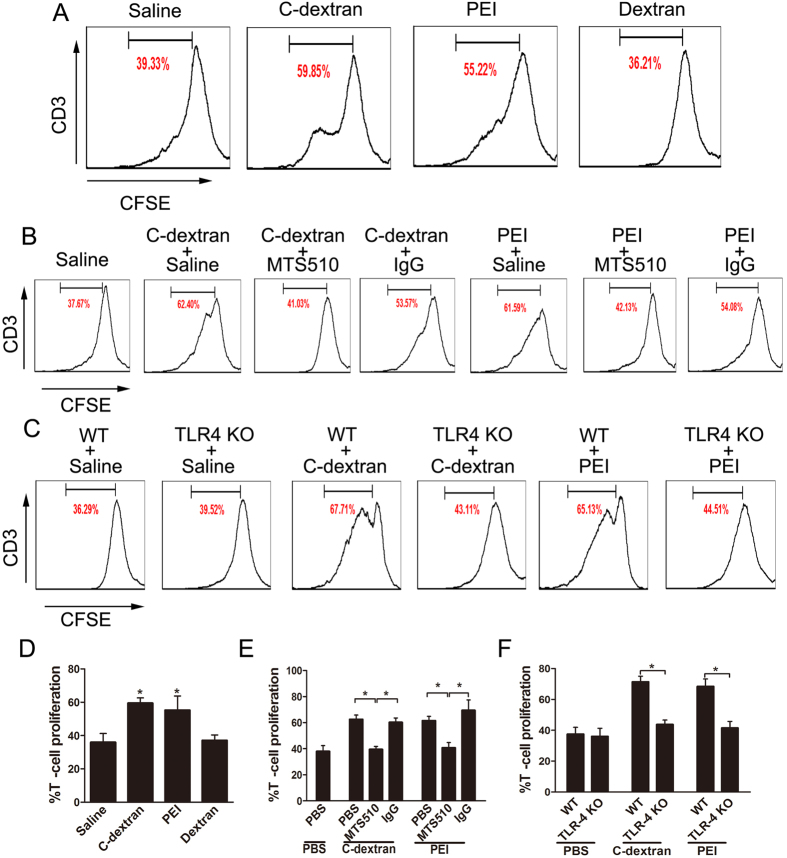
Effect of the cationic polymers on the immunosuppressive functions of tumour-infiltrating MDSCs. The proliferation of CD3+T cells was tested with the CFSE dilution assay, after the labelled splenocytes were co-cultured with the differently treated MDSCs (2:1) for 4 days in the presence of Con A: (**A,D**) MDSCs from tumour- bearing mice were treated with C-dextran, PEI or dextran (25 μg mL^−1^) for 6 hours and rinsed before co-culture; (**B**,**E**) MDSCs were pre-treated with PBS, TLR-4-neutralising antibody (MTS510) and control IgG for 1 hour, treated with C-dextran or PEI (25 μg mL^−1^) for 6 hours and rinsed before co-culture; (**C**,**F**) MDSCs from WT or TLR-4^−/−^ (KO) tumour-bearing mice were treated with C-dextran or PEI (25 μg mL^−1^) for 6 hours and rinsed before co-culture. The data represent 3–4 independent experiments (n* ***= **3–4, ^*^P ≤ 0.05 versus saline).

T cell proliferation assay suggested that, when treated with cationic materials, MDSCs lost their suppressive function and promoted T cells proliferation (Fig. 2A,D). However, this suppression was substantially relieved when TLR-4 signalling was blocked with either MTS510 (Fig. 2B,E) or disabled in TLR-4^−/−^ (KO) mice (Fig. 2C,F).

### The anti-cancer performance of cationic material

**Figure 3:**
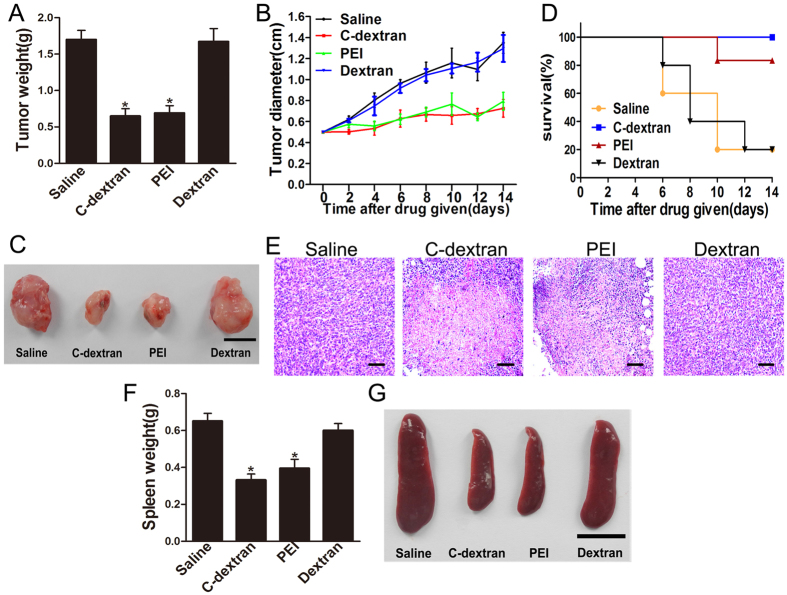
Effect of the cationic polymers on tumour growth *in vivo*. (**A**) The average tumour weight after the mice were treated with cationic polymers for 14 days; (**B**) tumour growth curves from mice that received intratumoural injection of saline, C-dextran, PEI or dextran. (**C**) Representative images of tumour samples harvested from animals in each treatment cohort (scare bar: 1 cm). (**D**) Survival rate of tumour-bearing mice treated with saline, C-dextran, PEI or Dextran. (**E**) H&E histological assessment of the tumour sections from mice that received saline, C-dextran, PEI or dextran (scale bar: 100 μm). (**F**) The mean spleen weights on day 14 after treatment with cationic polymers. (**G**) Representative images of spleens harvested from animals in each treatment cohort (scare bar: 1 cm; *n ***= **8–10 mice per group; ^*^P ≤ 0.05 versus saline).

Because cationic polymers could polarize MDSCs from M2- to M1-type, we examined whether this would affect tumour growth. In the mouse model, intratumoural administration of C-dextran and PEI reduced tumour weight (Fig. 3A) and size (Fig. 3B,C) and prolonged survival period (Fig. 3D). Notably, all mice in the group treated with C-dextran survived through the 14-day test period. Histologic analysis further revealed that C-dextran and PEI caused large areas of necrosis in tumour tissue, whereas saline or dextran showed little effect (Fig. 3E). These findings clearly suggest the anti-tumour effect of both cationic polymers used. In addition, both the weight (Fig. 3F) and size (Fig. 3G) of the spleen were decreased in the cationic polymer-treated groups, indicating the effect of these cationic polymers on the immune system.

### Cationic polymers re-polarize MDSCs towards M1-type

**Figure 4:**
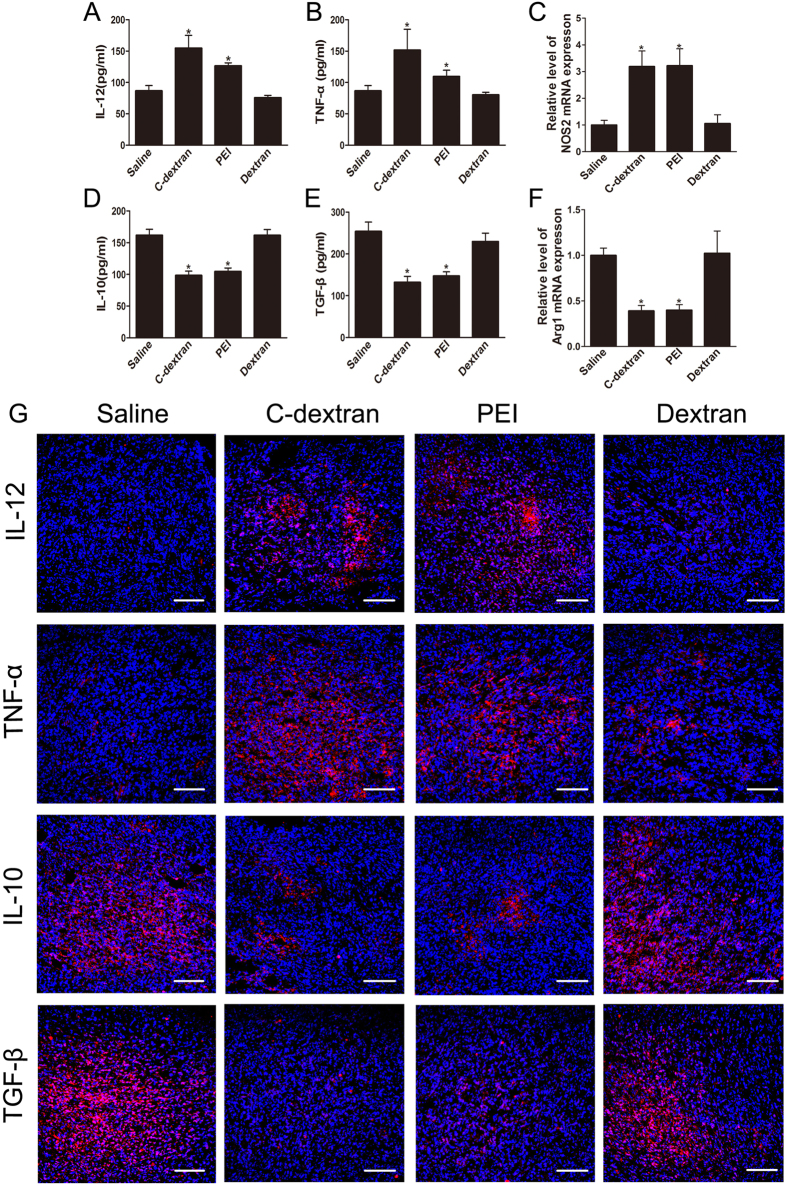
Effect of the cationic polymers on MDSC polarization *in vivo*. The concentrations of (**A**) IL-12, (**B**) TNF-α, (**D**) IL-10 and (**E**) TGF-β in the supernatant of MDSCs were determined by ELISA. The transcriptional levels of (**C**) iNOS2 and (**F**) Arg1 in MDSCs were analysed by qRT-PCR. (**G**) Immunofluorescent staining for IL-12, TNF-α, IL-10 or TGF-β (red), counterstained with DAPI (blue), in 4T1 tumour tissues from mice treated with saline, C-dextran, PEI or dextran. Scale bar, 100 μm. *n ***= **8–10 mice per group. Values are representative as the mean (SME) (*n ***= **8–10 mice per group; ^*^P ≤ 0.05 versus saline).

To examine whether the anti-tumour effect of cationic materials was related to change in the MDSC phenotype, we isolated MDSCs from these tumours and detected the levels of polarization markers of MDSCs. Tumour-infiltrating MDSCs treated with C-dextran and PEI secreted significantly higher levels of IL-12 (Fig. 4A) and TNF-α (Fig. 4B), but produced lower levels of IL-10 (Fig. 4D) and TGF-β (Fig. 4E) than the control group. These cationic polymers also increased the expression of NOS2 (Fig. 4C) while decreased that of Arg1 (Fig. 4F) at the transcriptional level. The results of immunofluorescent staining for IL-12, TNF-α, IL-10 and TGF-β were in good agreement with the ELISA outcomes (Fig. 4G), suggesting that the cationic polymers effectively induced a switch of MDSC into M1 polarization.

### Cationic polymers decrease MDSCs numbers in the blood, spleen, tumour and bone marrow (BM)

**Figure 5:**
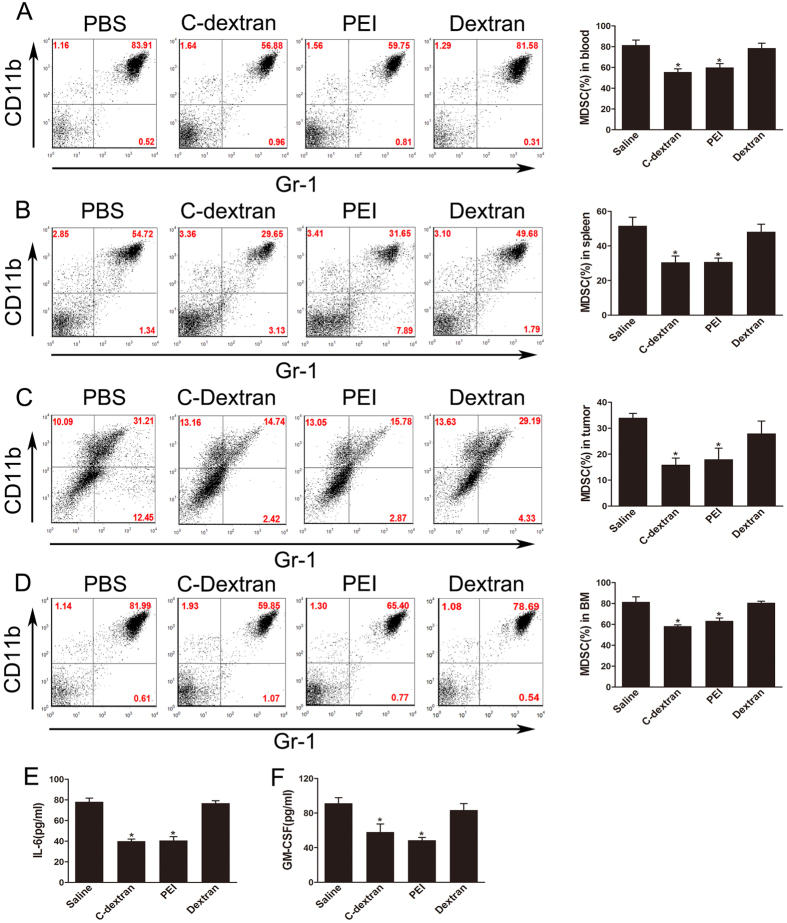
Effect of the cationic polymers on the survival of MDSC *in vivo*. (**A**) The proportion of MDSCs (right) and representative flow cytometry analysis (left) in blood after 14 days post treatment with cationic polymers; (**B**) The proportion of MDSCs (right) and representative flow cytometry analysis (left) in spleen from mice that received intratumoural injection of saline, C-dextran, PEI or dextran; (**C**) The proportion of MDSCs (right) and representative flow cytometry analysis (left) in tumour tissues after 14 days post treatment with cationic polymers. (**D**) The proportion of MDSCs (right) and representative flow cytometry analysis (left) in bone marrow (BM) after 14 days post treatment with cationic polymers. (**E,F**) The level of IL-6 and GM-CSF in the serum from tumour-bearing mice treated with the cationic polymers were measured by ELISA (*n ***= **8–10 mice per group; ^*^P ≤ 0.05 versus saline).

In both cancer patients and tumour-bearing mice, MDSCs are enriched in the blood, spleen and the tumour tissue. We tested whether administration of cationic polymers in 4T1 tumour-bearing mice would change this distribution. Following a two-week treatment, we found that both PEI and C-dextran significantly reduced the percentage of tumour-induced MDSCs in the blood (Fig. 5A), spleen (Fig. 5B), tumour (Fig. 5C) and particularly the bone marrow (Fig. 5D). Treatment with these cationic polymers also down-regulated the serum levels of tumour-derived factors such as IL-6 (Fig. 5E) and GM-CSF (Fig. 5F), further highlighting the potency of cationic materials in eliminating tumour-induced MDSCs.

### Cationic polymers restore the proliferation and functions of CD4^+^ and CD8^+^ T cells

**Figure 6:**
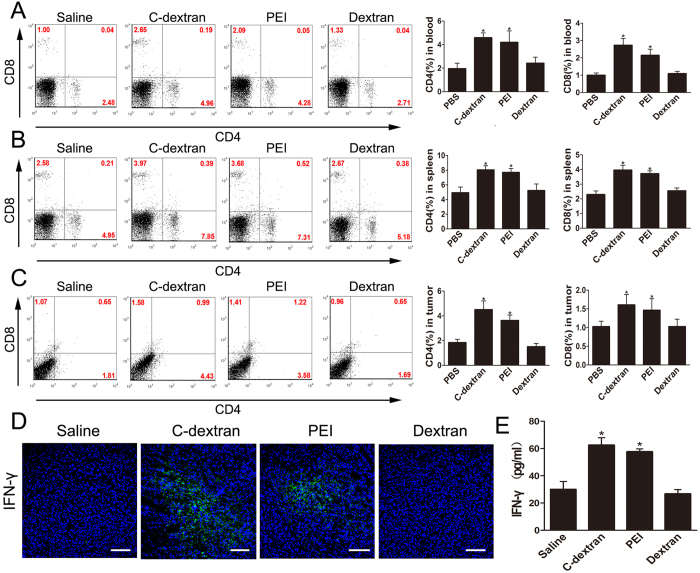
Effect of the cationic polymers on restoration of immunosurveillance against tumour *in vivo*. (**A–C**) The frequency of CD4+T cells and CD8+T cells (right) and representative flow cytometry analysis (left) in (**A**) the blood, (**B**) spleen and (**C**) tumour tissues after treatment with cationic polymers; (**D**) immunofluorescent staining for IFN-γ in the tumour tissues from mice treated with saline, C-dextran or PEI (scale bar; 100 μm); (**E**) Quantification of the IFN-γ level in the tumour tissues by ELISA (*n ***= **8–10 mice per group; ^*^P ≤ 0.05 versus saline).

Since we demonstrated above that the cationic polymers could eliminate and re-polarize MDSCs – which were well known to inhibit T cells, we asked whether these materials could restore the proliferation and function of T cells. First, we analysed the levels of CD4^+^ and CD8^+^ T cells in the blood, spleen and tumour tissues from different mice groups. We found that C-dextran and PEI increased the percentages of CD4^+^ and CD8^+^ T cells in the blood (Fig. 6A), spleen (Fig. 6B) as well as tumour tissues (Fig. 6C); while saline or dextran had no such effect. Next, we analysed the production of IFN-γ, which reflects the activation of T cells. The two cationic polymers clearly increased IFN-γ expression in the tumour tissue, according to both immunofluorescent staining (Fig. 6D) and ELISA test (Fig. 6E). These results suggest that both cationic polymers promote the proliferation and activity of CD4^+^ and CD8^+^ T cells in tumour-bearing mice.

### TLR-4 signalling is required for the anti-tumour activity of cationic polymers

**Figure 7:**
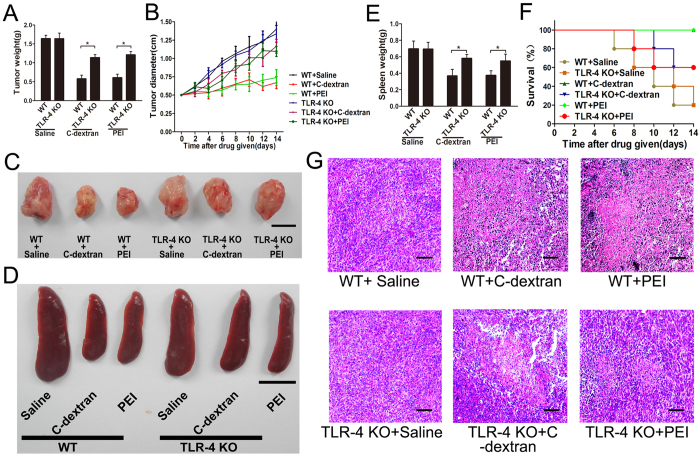
Effect of the cationic polymers on tumour growth in TLR-4^−/−^ (KO) mice. (**A**) The average tumour weight after the mice were treated with cationic polymers for 14 days; (**B**) tumour growth curves from mice that received intratumoural injection of saline, C-dextran or PEI; (**C,D**) representative images of (**C**) tumour and (**D**) spleen samples harvested from animals in each treatment cohort (scale bar: 1 cm); (**E**) the average spleen weight after the mice were treated with cationic polymers for 14 days; (**F**) survival rate of tumour-bearing WT or TLR-4^−/−^ mice treated with saline, C-dextran or PEI; (**G**) histological analysis (H&E) of the tumour sections from mice that received saline, C-dextran, PEI or dextran treatment (scale bar: 100 μm; *n ***= **8–10 mice per group; ^*^P ≤ 0.05 versus saline).

In line with the above findings that C-dextran or PEI failed to alter the phenotype of TLR-4^−/−^ (KO) MDSCs, we found that the anti-tumour effect of these cationic polymers was substantially attenuated in TLR-4^−/−^ (KO) mice. In these knockout mice, cationic polymers failed to suppress the tumour (Fig. 7A–C). No remedial effect was found in terms of the spleen size (Fig. 7D)/weight (Fig. 7E) or animal survival period (Fig. 7F) in TLR-4^−/−^ (KO) mice, in sharp contrast with the performance of these cationic polymers observed in WT mice. Histological staining further indicated mass necrosis in WT mice – but very little in TLR4^−/−^ (KO) mice – treated with the cationic polymers (Fig. 7G).

**Figure 8:**
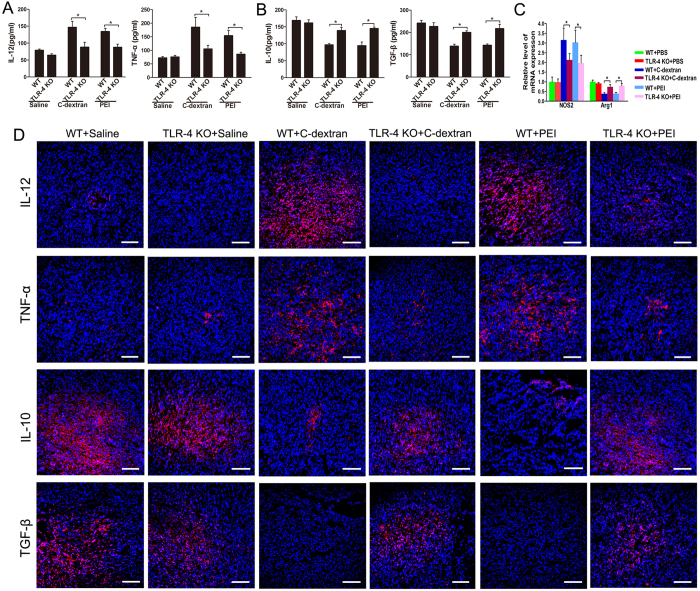
Effect of the cationic polymers on MDSC polarization in TLR-4^−/−^ (KO) mice. (**A,B**) The concentrations of (**A**) IL-12, TNF-α, (**B**) IL-10 and TGF-β in the supernatant of MDSCs from tumour-bearing WT or TLR-4^−/−^ mice were measured by ELISA; (**C**) transcriptional levels of iNOS2 and Arg1 in MDSCs after the treatment with cationic polymers; (**D**) immunofluorescent staining for IL-12, TNF-α, IL-10 and TGF-β (all red), counterstained with DAPI (blue), in the 4T1 tumour tissues collected from WT or TLR-4^−/−^ mice treated with saline, C-dextran or PEI (scale bar: 100 μm; *n ***= **8–10 mice per group; ^*^P ≤ 0.05 versus saline).

To further evaluate whether the cationic polymers would affect the MDSCs phenotype in the absence of TLR-4 signalling, we isolated MDSCs from TLR-4^−/−^ (KO) mice in each treatment cohort and analysed the levels of cytokines and related markers. In the TLR-4^−/−^ (KO) mice, C-dextran and PEI failed to increase the levels of M1 markers including TNF-α, IL-12 (Fig. 8A,D) and Nos2 (Fig. 8C) or to decrease those of M2 markers including IL-10, TGF-β (Fig. 8B,D) and Arg1 (Fig. 8C), though both cationic polymers exhibited these activities in WT mice. In summary, these data highlight that TLR4 signalling is necessary for the cationic polymers to exert their anti-tumour activities.

## Discussion

In this study, we demonstrated that re-polarizing MDSCs into a M1 type with two cationic polymers could effectively restore local immune surveillance and eventually inhibit tumour growth. Our results lend weight to the emerging findings that MDSCs, specifically the M2-polarized ones, play a key role in mediating immunosuppression in certain cancer types^2^,^10^,^14^,^30^,^31^. Our findings provide insights for design of new, polymer-based therapeutics for cancer immunotherapy.

In line with our previous report that PEI, a cationic polymer, could activate antigen-presenting cells (APCs) also via TLR signalling^32^, we found in this study that both cationic polymers of C-dextran and PEI exerted their *in-situ* anti-cancer effects through regulation of the immune system in a more comprehensive manner^33^. Firstly, as one of the most apparent evidence, treatment with either cationic polymer effectively decreased the weight of mice spleen. Secondly and more specifically, both polymers directly stimulated the expression of IL-12 and TNF-α in MDSCs. These ‘M1-representative’ cytokines, if appropriately harnessed, may have immediate impact against tumour – the former could enhance Th1 differentiation and stimulate the release of IFN-γ, serving as a potent adjuvant in cancer immune therapy;^34^ while the latter, a pro-inflammatory cytokine with complex biological function, could directly suppress certain tumours^35^. Thirdly, in addition to such MDSC-‘re-educating’ effects, both cationic polymers directly decreased the number of MDSCs and, in contrast, increased that of both CD4^+^ T and CD8^+^ T cells in the blood, spleen and the tumour tissues. Restoration of the T lymphocytic functions in the tumour environment proved crucial, as it efficiently induced massive necrosis in the tumour cells. To sum up, these cationic polymers exert an efficient and unique anti-cancer effect through a combination of immuno-regulatory mechanisms.

Besides cationic polymers, some other compounds, such as Docetaxel and curcumin, have been reported to polarize tumour-induced MDSCs from M2- towards M1-like phenotype and display anti-tumour activity, possibly through the inhibition of STAT3 phosphorylation (pSTAT3)^16^,^36^. In addition, numerous other therapeutic agents or biomolecules have also been employed to either convert MDSCs from M2- into M1-type MDSCs (such as paired immunoglobulin-like receptor B (PIR-B)^37^,^38^) or eliminate MDSCs (such as doxorubicin^39^), in an attempt to abolish MDSC-mediated immunosuppression and restore immune surveillance against tumour. These strategies, despite having achieved some positive outcomes, encounter substantial challenges^16^,^23^,^40^. For example, anti-Gr-1 antibody was capable of eliminating Gr-1^+^ MDSCs to restore T-cell tumouricidal activity, but it also eliminated the mature granulocytes and led to severe immunosuppression; in addition, it is unrealistic to apply this antibody to humans^41^. Doxorubicin, a representative chemotherapeutic agent, proved powerful in eliminating MDSCs but its use is substantially hindered by its lethal adverse effect of cardiotoxicity^42^. Additionally, CpG-containing oligonucleotides (ODN) showed promising potential in re-polarizing cultured macrophages, but it lacks *in vivo* stability^43^. In contrast, the cationic polymers are free of these concerns and exerted no toxicity to normal tissues in mice at the dose of administration. They are easy to prepare, modify and process into various forms of immunotherapeutic tools. These polymer-specific advantages add further values to the therapeutic potential of the cationic materials.

We demonstrated that the cationic polymer-induced MDSCs polarization require TLR-4 signalling. In general, TLRs recognize specific components of microbial origin and trigger inflammation, leading to both innate and adaptive immune responses against tumour^44^,^45^. Other TLR ligands have also been trailed for this purpose, with both negative and positive outcomes. For example, the aforementioned CpG ODN is a classic ligand of TLR9 with potent immuno-stimulatory activity to promote host response against cancer^46^. Although CpG ODN was also found to modulate the functions of TLR9-expressing MDSCs^23^,^47^, it could not directly activate MDSCs to stimulate anti-tumour cytokines^23^ and failed to prove efficacious in human clinical trials^48^,^49^. Meanwhile, TLR4 signalling plays an important role in MDSCs polarization. Lipopolysaccharide (LPS), a classic TLR4 ligand, could bind TLR4 and activate Erk, NF-κB and STAT1, consequently polarizing tumour-induced MDSCs into M1 type^14^. It is very likely that cationic polymers activate Erk, NF-κB and STAT1 to regulate the MDSCs phenotypes, since our present and previous studies have confirmed that these polymers, like LPS, are also ligands of TLR4^27^. Therefore, it would be interesting to devise therapeutics targeting TLR4 for the modulation of MDCS phenotypes. But meanwhile, TLR4 should not be the only receptor that mediated the effect of cationic polymers, because this effect was not completely abolished either in TLR4^−/−^ (KO) mice or using the TLR4-neutralising antibody. Further understanding of the interactions between cationic polymers and MDSCs (and other myeloid cell types) at the molecular level is required for better design of cancer immunotherapeutic approaches aimed at re-modelling the tumour microenvironment.

## Conclusion

In summary, we demonstrate a strategy to restore the local immunosurveillance in the tumour microenvironment by application of two readily-acquired cationic polymers, C-dextran and PEI, which re-polarize MDSCs from M2- to M1-type through TLR4-mediated signalling. These polymers proved highly efficacious in tackling immunosuppression and eradicating tumour in the 4T1 tumour-bearing mouse model. Our findings highlight the unique potential of bioactive polymers in interacting with the immune system and triggering desired immune responses. Further investigations into the biological functions of these polymers may provide insights for development of new, polymer-based immunotherapeutic tools.

## Methods

### Reagents and C-dextran preparation

PEI (25 kDa), dextran (70 kDa) and 1, 1′-carbonyldiimidazole (CDI) were purchased from Sigma (St. Louis, MO, USA). Other chemical reagents were purchased from Sangon Biotech (Shanghai, China) unless otherwise stated. We prepared cationic dextran (C-dextran) and determined the cationic degree with elemental analysis, following our previous study^50^.

### Mouse model

Animal protocols were reviewed and approved by the Animal Care and Use Committee of Nanjing University, and conformed to the *Guidelines for the Care and Use of Laboratory Animals* published by the National Institutes of Health.

BALB/c mice (female, 6–8 weeks old) and C57BL/10JScNJ mice (female, 6–8 weeks old) were purchased from the Animal Centre of Yangzhou University (Yangzhou, China). Toll-like receptor 4-deficient (TLR4^−/−^) mice were bred in-house using breeding pairs provided by The Model Animal Research Centre of Nanjing University (Nanjing, China). Animals were acclimatized in a ventilated, temperature-controlled room (23 °C) with a 12 h light/12 h dark cycle. All animals had free access to rodent chow and water and were treated in strict accordance with the institutional ethical regulation on animal experiments. Mouse mammary carcinoma cell line 4T1 was obtained from ATCC (CRL-2539^TM^). Cells were grown and maintained in RPMI-1640 medium supplement with 10% FBS. To generate the heterotopic tumour model, 1 × 10^6^ cells were injected subcutaneously into the left armpit of the animals. We measured the tumour size with calliper and weighted the tumour samples upon harvest.

### MDSC isolation and treatment

Mice were sacrificed and their spleens harvested when their tumour sizes exceeded 1.0 cm. After lysis of red blood cells (RBCs), splenocytes were fractionated with a Percoll (GE Healthcare, USA) density gradient as previously report^51^,^52^. Briefly, 0.5–1 × 10^8^ cells were re-suspended in 2 mL of 100% Percoll solution. Then, each of 70%, 60%, 50%, and 40% Percoll (2 mL) and HBSS (1 mL) were carefully layered over the cell suspension. After centrifugation at 1800 × g for 20 min, cells were collected from the gradient interfaces. Cells banding between 40% and 50% were labelled as fraction (Fr.) I; between 50% and 60% as Fr. II; and between 60 and 70% as Fr. III. Cells were collected from the gradient interface. The Fr. II cells were MDSCs. After washing, MDSC were subsequently cultured in RPMI-1640 medium containing 10% FBS. We confirmed both the purity and viability of MDSCs were greater than 90% with flow cytometry (CD11b; Gr-1) and trypan blue staining, respectively. To evaluate the effect of cationic polymers on MDSCs, we incubated the cells with dextran, C-dextran or PEI (25 μg mL^−1^) for 6 h. In blocking assays, we treated the cells with 20 μg/ml mouse TLR-4/MD-2 neutralizing antibody (MTS510, eBioscience), 20 μg/ml isotype control antibody (Rat IgG2a, Kappa) or PBS for 45 min before adding cationic materials.

### Anti-tumour activity of cationic polymers

The anti-tumour activity of cationic materials was examined in the subcutaneous mammary carcinoma 4T1-bearing mice. When the tumour size reached about 0.5 cm in diameter, the subcutaneous mammary carcinoma 4T1-bearing mice were injected intratumourally with dextran, PEI, C-dextran (1 mg kg^−1^ body weight) every two days. After the administration of the drugs, the tumour sizes were examined every two days. Fourteen days after the first administration of cationic polymers, all tumours and spleens were excised, weighed, and all tumour tissues were sectioned for histopathological and immunofluorescent analysis. MDSCs isolated from these tumours were used for identification of MDSCs markers by flow cytometry and analysis of secreted cytokines (IL-10, IL-12, TNF-α and TGF-β). To evaluate the overall survival, the tumour-bearing mice were given therapeutic agents intratumourally every two days after the size of tumour burden achieved about 0.5 cm. Once the tumour size reached 1.3 cm, the study was terminated and the mice were sacrificed.

### Blood, spleen and tumour tissues cells Preparation

Fourteen days after the first administration of cationic polymers, blood, spleen and intratumour cells were prepared from differently groups (every group has 8–10 mice) under the same conditions. Peripheral blood samples were collected into 1.5-ml tubes containing 3.8% sodium citrate anticoagulant. After lysis of red blood cells (RBCs), the cells were washed three times with ice-cold PBS containing 1% of BSA for flow cytometry analysis. For the spleen cells, spleens were collected in sterile HBSS without Ca^2+^ and Mg^2+^, grinded and filtered. After depleting erythrocytes, purified splenic cells were collected for flow cytometry analysis. For intratumoural immune cells, freshly resected solid tumours were cut into pieces and then enzymatically digested in HBSS containing 0.2% collagenase IV (wt/vol) and 0.1% DNase (wt/vol) (Sangon Biotech, China) with gentle stirring at 80 rpm for 45 min 37 °C. The resulting cell suspension was filtered through 70 μL Nylon cell strainers (Falcon, USA) and then centrifuged at 1000 rpm for 5 min. After lysis of red blood cells (RBCs), the cells were washed three times with ice-cold PBS containing 1% of BSA for flow cytometry analysis. We confirmed the viability of these cells to be greater than 90% with trypan blue staining. To analyse the change of immune cells in blood, spleen and tumour after the cationic polymers treatments by the flow cytometry, we gated the total cells from the blood, spleen and tumour tissues.

### Quantitative real-time PCR (qRT-PCR)

Total RNA was extracted by using TRIzol reagent (Invitrogen) and checked for purity and concentration with A_260_/A_280_ reading (Biophotometer Plus, Eppendorf, Germany), before being reversely transcribed to cDNA for qRT-PCR experiments (ABI 7300). The primers used were below: NOS2 sense: 5′-CCAAGCCCTCACCTACTTCC-3′; antisense: 5′-CTCTGAGGGCTGACACAAGG-3′; Arg1 sense: 5′-CTCCAAGCCAAAGTCCTTAGAG-3′; antisense: 5′-AGGAGCTGTCATTAGGGACATC-3′; β-actin sense: 5′-GGTGTGATGGTGGGAATGGG-3′; antisense: 5′-ACGGTTGGCCTTAGGGTTCAG-3′.

### Immunofluorescent staining

Frozen tumour tissue was sectioned and incubated with the following primary antibodies: rabbit anti-mouse IFN-γ (BioLegend), rabbit anti-mouse IL-10, rabbit anti-mouse IL-12, rabbit anti-mouse TNF-α, rabbit anti-mouse TGF-β (Boster) at 4 °C for overnight. The sections were washed with Tris-buffer saline and subsequently stained with the following secondary antibodies: Alexa 488 labelled donkey anti-hamster, Alexa 546 labelled donkey anti-rabbit (Life technologies), with the nuclei being counterstained with 4′, 6-diamidino-2-phenylindole (DAPI). Samples were imaged using a Nikon confocal microscope. Each staining was performed with three parallel samples and samples stained with secondary antibodies alone were set to determine the background threshold.

### Flow cytometry analysis

Flow cytometry was performed using a FACSCalibur device (Becton-Dickinson) and assessed with FCS express V3. After washing with PBS, 1 × 10^5^ cells from spleen, blood or tumour tissues were blocked in 100 μL 1% BSA at 4 °C for 30 min and then incubated with antibodies for CD11b, Gr-1, CD3, CD4 or CD8 (BioLegend), IL-12 and TNF-α (eBioscience) for another 30 min at 4 °C.

### MDSC suppression assay

For the *in vitro* suppression assay, total spleen cells were isolated from healthy mice followed by red blood cell lysis. These cells were labelled with CFSE (Beyotime), cultured in a 96-well plate (1 × 10^5^ cells per well), and stimulated with Con A (4 μg mL^−1^, Sigma-Aldrich). MDSCs were incubated with dextran, C-dextran or PEI for 6 hours. Then, the CFSE-labelled spleen cells were co-cultured with MDSCs at a 2:1 ratio. After 4 days, CFSE dye dilution after CD3 staining was analyzed with flow cytometry. In doing so, we assessed whether the polymers would affect the suppressive effect of MDSCs on T cell proliferation.

### ELISA analysis

The concentrations of IL-12, TNF-α, IL-10, TGF-β, IL-6 and GM-CSF in cell-culture supernatant were measured by using corresponding ELISA kits (eBioscience), following the manufacturer’s instructions.

### Statistical Analysis

The data are presented as the mean ± S.E.M. Differences between multiple groups were compared using one-way ANOVA (GraphPad Prism 5). The significance level was considered statistically at P **≤ **0.05, and P **≤ **0.01 indicated a strongly significant difference.

## Additional Information

**How to cite this article**: He, W. *et al.* Re-polarizing Myeloid-derived Suppressor Cells (MDSCs) with Cationic Polymers for Cancer Immunotherapy. *Sci. Rep.* **6**, 24506; doi: 10.1038/srep24506 (2016).

## Supplementary Material

Supplementary Figure
