# Supplementary material for: Re-polarizing Myeloid-derived Suppressor Cells (MDSCs) with Cationic Polymers for Cancer Immunotherapy
Source: Sci Rep. 2016 Apr 14;6:24506. doi: 10.1038/srep24506 (PMC4830950; doi:10.1038/srep24506)
Supplement: Supplementary Figure [file srep24506-s1.doc]

Supplementary figure

Re-polarizing Myeloid-derived Suppressor Cells (MDSCs) with Cationic Polymers for Cancer Immunotherapy

Wei He^1^, Pei Liang^1^, Guangxing Guo^1^, Zhen Huang^1^, Yiming Niu^2^, Lei Dong^1^, Chunming Wang^2^, Junfeng Zhang^1,3^

^1^ State Key Laboratory of Pharmaceutical Biotechnology, NJU Advanced Institute for Life Sciences (NAILS), School of life sciences, Nanjing University, 163 Xianlin Avenue, Nanjing 210093, China.

^2^ State Key Laboratory of Quality Research in Chinese Medicine, Institute of Chinese Medical Sciences, University of Macau, Taipa, Macau SAR.

^3^ Jiangsu Provincial Laboratory for Nano-Technology, Nanjing University, Nanjing, China

Correspondence and requests for materials should be addressed to L.D. (email: [leidong@nju.edu.cn](mailto:leidong@nju.edu.cn)), C.W. (email: [cmwang@umac.mo](mailto:cmwang@umac.mo)), or J.Z. (email: [jfzhang@nju.edu.cn](mailto:jfzhang@nju.edu.cn))


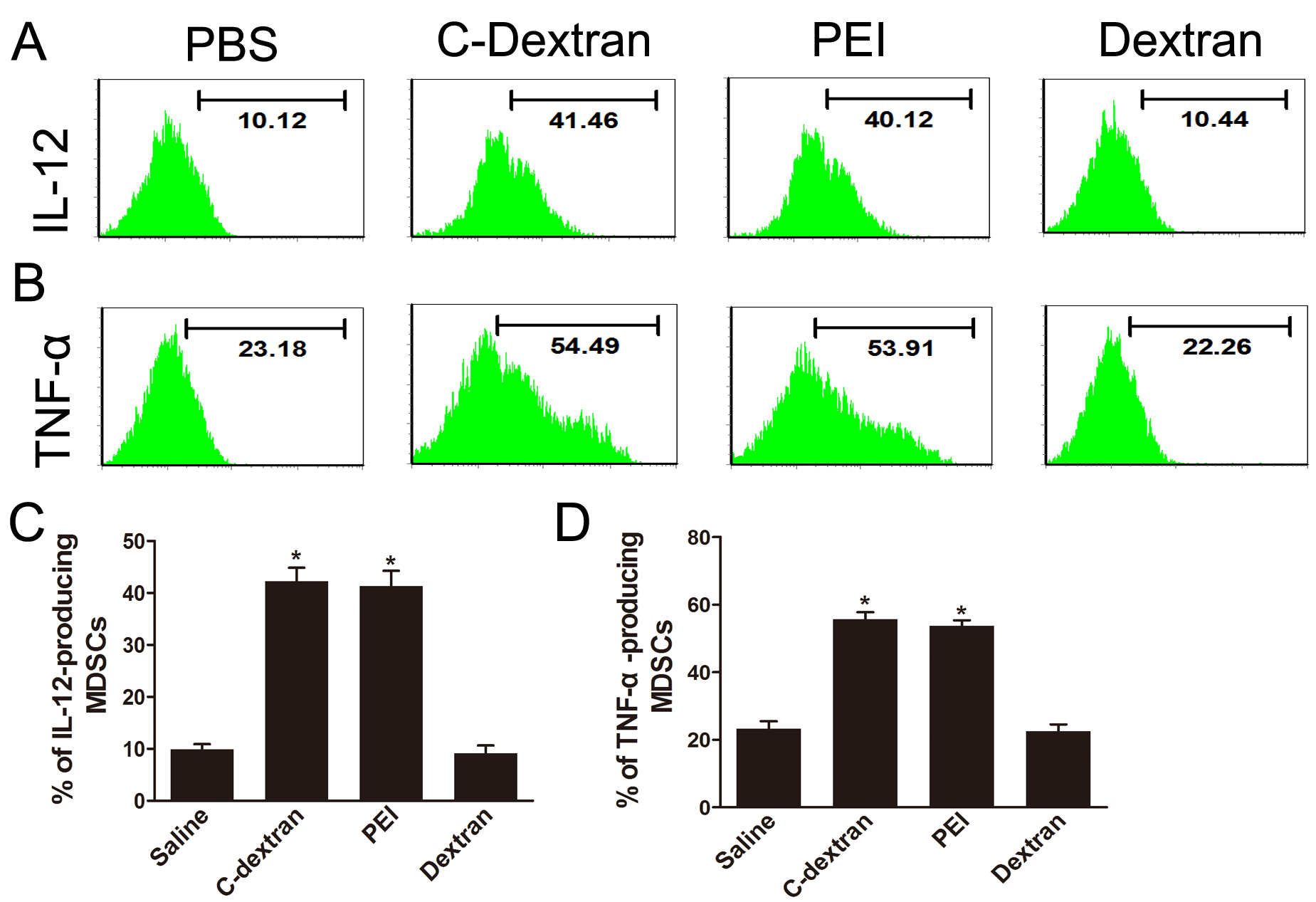


**Figure S1**. The effects of cationic polymers on the polarization of MDSCs. (A and B) Representative charts from flow cytometry analysis and (C and D) the proportion of MDSCs expressing IL-12 (A and C) and TNF-α (B and D) after the treatment of PBS, C-dextran, PEI or dextran.
